# Progress on the efficacy and mechanism of action of panax ginseng monomer saponins treat toxicity

**DOI:** 10.3389/fphar.2022.1022266

**Published:** 2022-09-19

**Authors:** Xinyi Wang, Rongcan Wang, Yongfei Qiao, Yali Li

**Affiliations:** ^1^ Institute of Special Animal and Plant Sciences of Chinese Academy of Agricultural Sciences, Changchun, China; ^2^ Jilin Provincial Key Laboratory of Traditional Chinese Medicinal Materials Cultivation and Propagation, Changchun, China

**Keywords:** ginsenosides, exogenous toxicity, Panax ginseng monomer saponins, treatment, mini-review

## Abstract

As a traditional Chinese herbal medicine, *Panax ginseng* C. A. Meyer (PG) has preventive and therapeutic effects on various diseases. Ginsenosides are main active ingredients of PG and have good pharmacological effects. Due to the diversity of chemical structures and physicochemical properties of ginsenosides, Currently, related studies on PG monomer saponins are mainly focused on the cardiovascular system, nervous system, antidiabetic, and antitumor. There are few types of research on the toxin treatment, predominantly exogenous toxicity. PG and its monomer ginsenosides are undoubtedly a practical option for treating exogenous toxicity for drug-induced or metal-induced side effects such as nephrotoxicity, hepatotoxicity, cardiotoxicity, metal toxicity and other exogenous toxicity caused by drugs or metals. The mechanism focuses on antioxidant, anti-inflammatory, and anti-apoptotic, as well as modulation of signaling pathways. It summarized the therapeutic effects of ginseng monomer saponins on exogenous toxicity and demonstrated that ginsenosides could be used as potential drugs to treat exogenous toxicity and reduce drug toxicities.

## Introduction

*Panax ginseng* C. A. Meyer (PG) has been used worldwide as a traditional medicine for thousands of years; numerous studies have shown that ginsenosides are the main active ingredients of PG. Currently, more than 150 natural ginsenosides have been isolated and identified from various parts of ginseng herbs (Christensen, 2009). According to the different skeletons of ginsenoside glycosides, ginsenosides can be divided into three saponins, protopanxdiol, protopanaxtriol and oleanolic acid (Wang et al., 2006). There are numerous reports on the biological effects of ginsenosides, such as immune enhancement (Bai et al., 2019), hepatoprotection (Fan et al., 2019), neuroprotection (Ying et al., 2019), anti-inflammatory (Chen et al., 2021), anti-tumour (Liu and Fan, 2019), and ginsenosides also reverse the drug resistance of tumour cells caused by other chemotherapy drugs (Meng et al., 2019). Different ginsenoside monomers have different functions. For example, ginsenoside Rh2 can inhibit the metastasis of cancer cells to other organs (Shi et al., 2014); ginsenoside Rg1 has the effect of excitation of the central nervous system and inhibition of platelet agglutination (Sun et al., 2016); ginsenoside Rg3 can inhibit the synthesis of proteins and adenine nucleoside triphosphate (ATP) in cancer cells during mitotic prophase and delay the proliferation and growth of cancer cells (Jiang et al., 2017).

Many western drugs are effective in the therapy treatment of diseases and play an essential role in the clinical management of tumors in particular, but their limitations are also evident; for example, the commonly used oncological drug Doxorubicin (DOX) causes cardiotoxicity. The items that contacted by us in our daily life may also contain toxic ingredients, for example, Trimethyltin in plastic stabilizers can cause neurotoxicity, and the presence of toxicity in metals such as iron and aluminum can also induce disease. Therefore, it is crucial to discover drugs from natural plants to mitigate drug toxicity and treat exogenous toxicity.

This paper reviewed the effects of ginsenosides on the treatment of exogenous toxins, and we hope that this review will lay the foundation for an in-depth study of biochemical mechanisms and pharmacological impact of ginsenosides and provide a reference for further development and utilization of ginsenosides in the treatment of exogenous toxicity.

## Effects of ginsenosides on cardiotoxicity

Tumours and cardiovascular diseases have become the top two causes of death in China’s urban population (Ma et al., 2020). In addition, it has been reported in the literature that the two disciplines are cross-cutting and that some long-term surviving malignant tumour patients may eventually die from heart disease rather than tumour, and heart disease has become the leading cause of non-cancer-related death in tumour patients (Zaorsky et al., 2017). In clinical practice, some antitumor therapies can also cause cardiotoxicity. Anthracyclines, alkylating agents, 5-FU and paclitaxel, are common chemotherapeutic drugs with cardiotoxicity, which can cause cardiovascular diseases such as heart failure, coronary artery lesions, hypertension and thrombosis (Luis Zamorano et al., 2016). Although there are many drugs for clinical use in oncology, current information on the exogenous cardiotoxic effects of ginsenosides for the treatment of the heart is focused on the cardiotoxicity caused by DOX and Trastuzumab (TZM).

Although DOX is an anthracycline antibiotic with powerful anti-tumor effects, it causes cumulative and dose-dependent cardiotoxicity, which leads to an increased risk of death in cancer patients. Thus, its clinical application is limited (Zhao et al., 2018, 2; Rawat et al., 2021). Ginsenosides protect the heart from various cardiovascular diseases by regulating multiple cellular signaling pathways. Ginsenoside Rg1 ameliorates DOX-induced cardiac insufficiency by inhibiting endoplasmic reticulum stress and autophagy (Xu et al., 2018). Rg1 increases phosphorylation of Akt and Erk, increases the ratio of Bcl-2 and Bax, and reduces cytochrome c release in mitochondria, thereby protecting the heart from DOX-induced apoptosis (Zhu et al., 2017). Ginsenoside Rg2 attenuates DOX-induced apoptosis by upregulating Akt phosphorylation and inhibiting p53 expression through the PI3K/Akt pathway in cardiomyocytes (Qiu et al., 2021). In summary, Rh2 may become a new protective agent in the clinical application of DOX (Wang H. et al., 2012).

Similarly, ginsenosides can achieve cardioprotective effects by regulating autophagy. Rb1 attenuates DOX-induced reduction in cardiomyocyte viability and inhibits the increase in autophagy-related structures, the conversion of light chain 3-I to light chain 3-II, and the reduction in p62 protein expression (Li et al., 2017). In addition, endoplasmic reticulum stress is another cause of cardiac dysfunction, closely associated with autophagy activation (Rashid et al., 2015). Echocardiographic and pathological findings suggest that ginsenoside Rg1 can significantly reduce DOX -induced cardiotoxicity. Endoplasmic reticulum stress and inhibition of autophagy may be the mechanism by which Rg1 ameliorates DOX-induced cardiac dysfunction (Xu et al., 2018).

TZM is a standard clinical treatment for breast cancer, but it has significant cardiotoxicity (Koulaouzidis et al., 2021). Rg2 induces autophagy in human cardiomyocytes (HCMs) by upregulating the expression levels of (p)-Akt, p-mTOR, beclin 1, light chain 3 (LC3) and autophagy protein 5 (ATG5), thereby treating TZM-induced cardiotoxicity (Liu et al., 2021). Liu et al. (2022) suggested that Rg2 could inhibit TZM-induced cardiac cytotoxicity. The mechanism might be related to the downregulation of proapoptotic proteins caspase-3, caspase-9, and BAX expression, which inhibited TZM-induced apoptosis in cardiac myocytes.

## Effects of ginsenosides on neurotoxicity and brain toxicity

### Ginsenosides attenuate neurotoxicity

Trimethyltin (TMT) is a by-product of the production of plastic stabilizers. It has been found in domestic water supplies, aquatic specimens, and marine environments (Gomez et al., 2007). TMT is a toxic organotin compound which selectively induces neurodegeneration in the limbic system, especially prominent in the hippocampus (Lee et al., 2016). Ginsenoside Re can against TMT-induced neurotoxicity through the PI3K/Akt signalling pathway of IL-6 (Tu et al., 2017). Another study discovered that Rg3 and Rh2 treat TMT-induced neurodegeneration by reducing oxidative stress and neuroinflammatory neurotoxicity (Hou et al., 2018). Hou et al. (2017) administered a single injection of 2 mg/kg body weight of TMT to ICR mice after pretreating them with ginsenoside Rd. Compared with saline-treated controls, Rd was found to act as a neuroprotective agent to prevent TMT-induced neurotoxicity. Cadmium (Cd) is a toxic and non-essential element for humans, which enters and accumulates in organisms through occupational exposure, contaminated air, water and food (Huang et al., 2017), Ren et al. (2021) reported that Rg1 eliminated Cd-induced toxicity and restored oxidative stress and inflammatory responses, and accordingly restored behavioural performance in animals, suggesting that Rg1 has an eliminating effect on Cd-induced neurotoxicity.

### Ginsenosides protect the brain from β-amyloid-induced toxicity

β-Amyloid (Aβ) aggregates cause complex neurotoxicity and play a vital role in the progression of Alzheimer’s disease (AD) (Ho et al., 2015). Prevention of Aβ-induced toxicity could lead to drug development for Alzheimer’s disease. In a double-transgenic AD mouse experiment, Yun et al. (2022) found that ginsenoside F1 exerts its beneficial effects by increasing insulin-degrading enzyme (IDE) and neprilysin (NEP) expression, providing scientific evidence regarding the applicability of Aβ treatment in AD patients. It has been suggested that Rb1 is likely to protect neurons from Aβ toxicity through the antioxidant pathway (Qian et al., 2009). Xie et al. (Xie et al., 2010) pretreated cells with Rb1 for 24 h and then added Aβ25-35 to the medium for another 24 h. They found that Rb1 pretreatment inhibited Aβ-induced Reactive oxygen species (ROS) overproduction and lipid peroxidation, increased the Bcl-2/Bax ratio, and attenuated caspase-3 activation, thereby increasing cell survival and protecting against Aβ-induced cell damage.

## Effects of ginsenosides on metal-induced toxicity

Iron accumulation is thought to be involved in the pathogenesis of Parkinson’s disease (PD) (Jiang et al., 2007). Several studies have shown that selectively high iron levels and oxidative stress due to elevated iron levels in the substantia nigra pars compacta (SNpc), play a crucial role in developing PD (Youdim et al., 2004, 28; Zecca et al., 2008). Rg1 reduces cellular iron accumulation and attenuates the inappropriate upregulation of divalent metal transporter 1 with the iron-responsive element (DMT1 + IRE) through the IRE/Iron regulatory protein (IRP) system to achieve neuroprotective effects against iron toxicity (Xu et al., 2010a). In addition, it was found that Rg1 could reduce iron influx and iron-induced oxidative stress by inhibiting the upregulation of DMT1-IRE(Xu et al., 2010b). Wang et al. (Wang et al., 2009) suggested that the neuroprotective effect of Rg1 on dopaminergic neurons against 1-Methy-4-phenyl-1,2,3,6-tetrahydropyridine (MPTP) is due to the ability to reduce nigrostriatal iron levels, which is achieved by regulating the expression of divalent metal transporter 1 (DMT1) and Ferroportin1 (FP1).

Aluminum (Al)-induced disorders of bone metabolism are a significant cause of osteoporosis. The research concluded that Rb1 significantly reverses osteoblast viability and osteoblast growth regulators, inhibits oxidative stress, and attenuates histological damage to osteoblasts by AlCl3(Zhu et al., 2016), activating the TGF-β1/Smad signaling pathway is one of the mechanisms by which Rg3 alleviates Al-induced bone damage (Song et al., 2021). Song et al. (2020) reported that Rg3 effectively alleviated AlCl3-induced osteoporosis by increasing the mRNA expression of transforming growth factor-β1, bone morphogenetic protein-2, insulin-like growth factor I, and core binding factor α1 to promote growth regulators and attenuate Al accumulation.

## Effects of ginsenosides on hepatotoxicity

Frequent overdose of acetaminophen (APAP) is one of the most common and essential triggers of acute hepatotoxicity (Xu et al., 2017). Ginsenoside Rg5 exerts hepatoprotective effects against APAP-induced acute hepatotoxicity. Wang et al. (2017) administered Rg5 to mice and found the protein expression of proliferating cell nuclear antigen (PCNA), Bax, cytochrome c, caspase-3, caspase-8, and caspase-9 was significantly inhibited in the Rg5 group compared with the control group. In contrast, the expression level of Bcl-2 protein was increased, indicating that Rg5 has anti-apoptotic ability in APAP-induced hepatotoxicity. Rk1 pretreatment significantly reduced serum alanine aminotransferase, aspartate aminotransferase, tumour necrosis factor, and interleukin-1β levels and significantly reversed APAP-induced liver tissue necrosis (Hu et al., 2019). Rb1 exhibits significant hepatoprotective effects against APAP-induced ALI by modulating MAPK and PI3K/Akt signalling pathway-mediated inflammatory responses (Ren et al., 2019). Rg3 exerts hepatoprotective effects on APAP-induced hepatotoxicity by inhibiting oxidative stress and inflammatory responses (Zhou et al., 2018).

Cisplatin (CP) is an effective antitumor drug widely used in cancer treatment, and hepatotoxicity is one of its side effects (Taghizadeh et al., 2021). Rg1 effectively prevents cisplatin-induced hepatotoxicity, mainly by inhibiting the binding of Keap1 and Nrf2, partly through the accumulation of p62 (Gao et al., 2017).

## Effects of ginsenosides on nephrotoxicity

Nephrotoxicity is a common side effect of chemotherapy and drugs. For example, the commonly used drug CP may cause severe nephrotoxicity, including tubular injury and renal failure (Miller et al., 2010; Manohar and Leung, 2017). Numerous studies have demonstrated that ginsenosides can promote the recovery of kidney function by regulating inflammation apoptosis and reducing kidney damage (Baek et al., 2006; Park et al., 2015; Wang et al., 2018). In CP mice, Rh2 treatment significantly increased the expression of Bcl-2. It decreased the expression of p53, Bax, cytochrome c, caspase-8, caspase-9, and caspase-3 in renal tissues, suggesting that Rh2 prevents CP-induced nephrotoxicity by acting on the cystein-mediated pathway (Qi et al., 2019). The increase in the percentage of apoptotic LLC-PK1 cells induced by CP treatment was also significantly reduced after Rh3 treatment (Lee and Kang, 2017). Thus, ginsenosides are potential agents for treating CP-induced nephrotoxicity (Table 1).

**TABLE 1:** Therapeutic effect of ginsenosides on CP-induced nephrotoxicity.

Ginsenosides	Experimental model	Dosage and method	Mechanisms	Effects	References
Rb3	ICR mouse	10 and 20 mg/kg by oral gavage	(↓) p62, ATG3, ATG5, ATG7, p-mTOR, the ratio of LC3-I/LC3-II	Rb3 regulates AMPK-/mTOR-mediated autophagy and inhibits apoptosis *in vitro* and *in vivo*, thereby alleviating CP-induced nephrotoxicity	Xing et al. (2019)
Rh2	Male SPF grade ICR mice (22–25 g)	20 and 40 mg/kg by gavaged (P.O)	(↑) Bcl-2	Rh2 protects against CP-induced nephrotoxicity by acting on caspase-mediated pathways	Qi et al. (2019)
			(↓) p53, Bax, cytochrome c, caspase-8, caspase-9, and caspase-3		
Re	Male ICR mice	25 mg/kg by oral gavage	(↓) Renal dysfunction, inflammatory cytokines, apoptosis, malondialdehyde in the kidney	The renal protective potential of Re may be partly related to its antioxidant, anti-inflammatory and anti-apoptotic effects	Wang et al. (2018)
Rh3	pig kidney epithelium, CL-101		(↓) JNK, ERK, p38, caspase-3, Proportion of apoptotic cells in LLC-PK1	Inhibition of JNK and ERK mitogen-activated protein kinase signaling cascade plays an important role in the renoprotective effects of Rh3	Lee and Kang, (2017)
Rg5	Male ICR mice (6–8 weeks old)	10 and 20 mg/kg administered intragastrically	(↑) Bcl-2	Rg5 attenuates CP-induced nephrotoxicity by reducing oxidative stress, inhibiting inflammation, and suppressing apoptosis in CP-treated mice	Li et al. (2016)
			(↓) NF-κB p65, COX-2, Bax		

ATG3, autophagy related three; ATG5, autophagy related five; ATG7, autophagy related seven; LC3-I, light chain 3-I; LC3-II, light chain 3-II; JNK, c-Jun N-terminal kinase; ERK, extracellular signal-regulated kinase; LLC-PK1, porcine renal proximal epithelial tubular; NF-kB, nuclear factor-kappa B; COX-2, cyclooxygenase 2.

## Effects of ginsenosides on reproductive toxicity

The value of ginsenosides in reproductive function was demonstrated in several reports. Many studies have shown that bisphenol A (BPA) can cause reproductive toxicity. Wang et al. (Wang L. et al., 2012) showed that ginsenosides (75 μg/ml) significantly inhibited the decrease in cell viability and increase in apoptosis inhibited by BPA through *in vitro* cell culture model experiments. These effects are mediated by preventing ERK1/2 phosphorylation and enhancing cellular antioxidant capacity. Testicular toxicity is one of the side effects of chemotherapeutic drugs. Ji et al. (2007) reported the therapeutic and preventive effects of protopanaxatriol saponin (PT) on the testicular organs of male mice under toxicity induction of the chemotherapeutic drug busulfan, and the damage to spermatogenic tubules in mice injected with PT was less than that of busulfan treatment alone. These results suggest that PT is effective in recovering male reproductive organs and overcomes the toxicity of busulfan. PT may be indicated for recovering male infertility caused by azoospermia and oligospermia. Endometriosis (EMS) is an estrogen-dependent gynaecological disorder, impaired NK cell cytotoxic activity is associated with clearance obstruction of ectopic endometrial tissue in the abdominal and pelvic cavity. Zhang et al. (2018) reported that PPD-pretreated ectopic endometrial stromal cells (eESCs) enhanced the cytotoxic activity of NK cells against eESCs, reduced the number of ectopic lesions and inhibited the growth of ectopic lesions in a mouse EMS model. They suggest that this effect may be through limiting estrogen-mediated autophagy regulation and enhancing NK cell cytotoxicity.

## Summary and observations

Due to the mutual influence and restriction of various saponin monomers, the unique medicinal and health-care properties of various monomeric saponins cannot be displayed, which significantly reduces the application value. In recent years, researchers at home and abroad have devoted themselves to the use of biological methods to produce ginsenoside products and have made corresponding progress and breakthroughs in biotechnology in various research fields such as tissue culture, transgenic plants, biosynthetic pathways and synthetic biology, which have laid an essential foundation for the preparation or production of ginsenoside products in large quantities. Most ginsenoside monomers on the market are relatively cheap, such as Rb1, Re, etc. The average price is about 0.289 USD/mg; individual monomeric saponins are rarer and more costly, with an average price of about 5.797 USD/mg.

**FIGURE 1:**
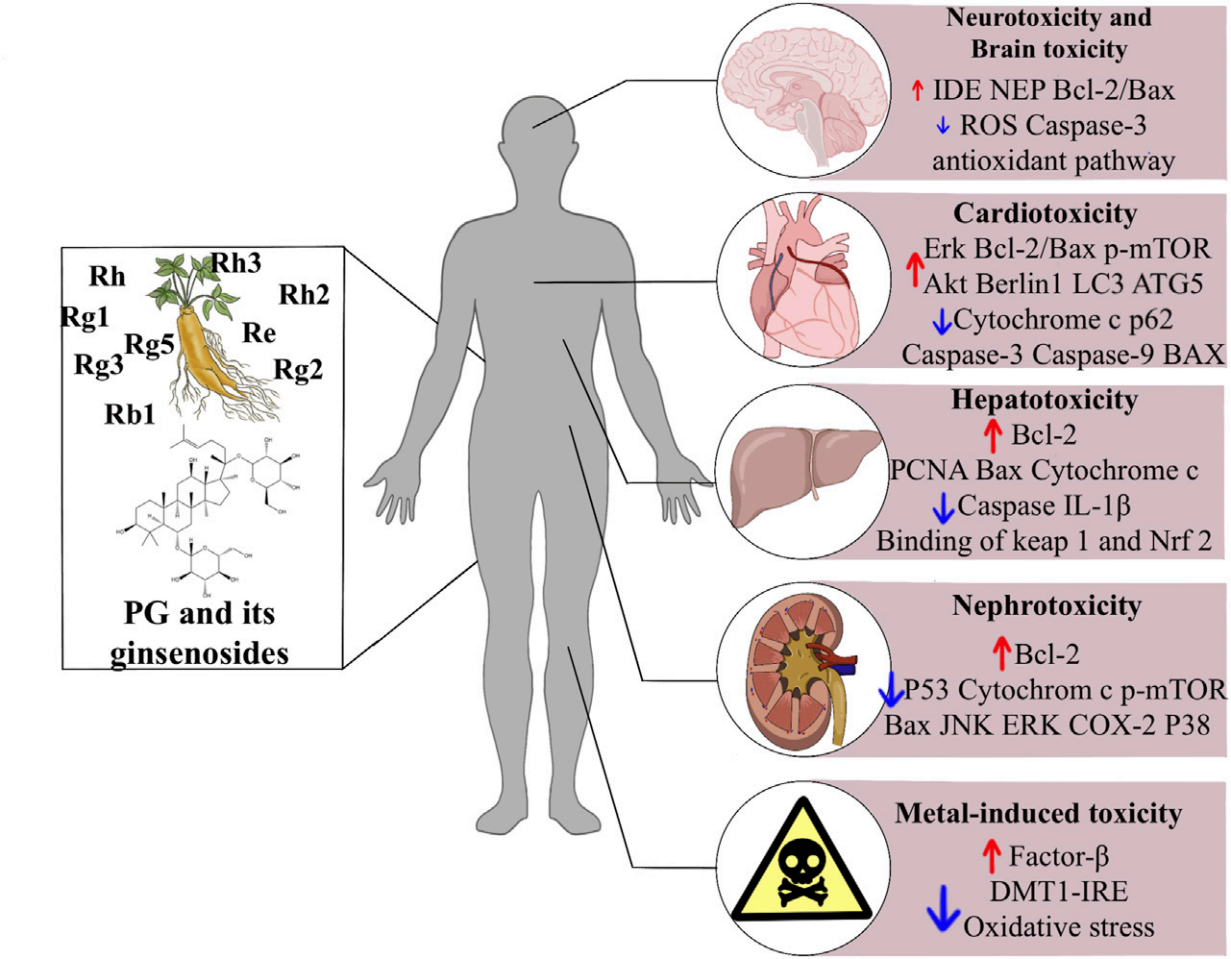
The effects of ginsenosides on the treatment of exogenous toxins. Abbreviations: ROS, reactive oxygen species; Erk, extracellular signal-regulated kinases; mTOR, mammalian target of rapamycin; LC3, light chain three; ATG5, autophagy related five; IL-1b, interleukin-1b; Nrf2, nuclear factor erythroid related factor 2; ATG3, autophagy related three; JNK, c-jun N-terminal kinase; ERK, extracellular signal-regulated kinase; COX-2, cyclooxygenase two; DMT1 + IRE, divalent metal transporter 1 with iron responsive element.

This review briefly summarizes the therapeutic potential of ginsenosides in drug toxicities and exogenous toxins, and explains the mechanism of action (Figure 1). Ginsenosides play an essential role in treating drug toxicity, especially in treating cancer patients, and can reduce drug toxicity and improve the survival quality of cancer patients after the cure. The underlying mechanism may be related to an increase in antioxidant enzymes and anti-apoptotic, anti-inflammatory signalling and immunostimulatory factors, as well as a decrease in pro-apoptotic, pro-inflammatory, immunosuppressive and pro-oxidant indices. Further studies revealed that this mechanism involves several signalling pathways, such as the classical antioxidant pathway: P62/KEAP1/NRF2, the apoptosis-related pathway: JNK/P53/CASPASE3, and the AMPK/mTOR signalling pathway, which plays a crucial role in the development of autophagy. In addition, ginsenosides play an essential role in the treatment of metal toxicity and the accumulation of toxins that may be caused by chemicals added to household products. In conclusion, ginsenosides are potential drugs for preventing and treating exogenous toxins.
